# Complete mitochondrial genome of *Eurhaphidophora fossa* (Orthoptera: Rhaphidophoridae: Rhaphidophorinae)

**DOI:** 10.1128/mra.01177-25

**Published:** 2026-04-23

**Authors:** Wenjing Wang, Tingting Yu, Xun Bian, Bin Zhang

**Affiliations:** 1 College of Life Sciences and Technology, Inner Mongolia Normal University 71203 , Hohhot, China; 2 Guangxi Key Laboratory of Rare and Endangered Animal Ecology, Guangxi Normal University 12388, Guilin, China; 3 Key Laboratory of Biodiversity conservation and Sustainable utilization for College and University of Inner Mongolia Autonomous Region, Hohhot, China; University of California Riverside, Riverside, California, USA

**Keywords:** mitogenome, Rhaphidophoridae, Rhaphidophorinae

## Abstract

We present the first complete genome of *Eurhaphidophora fossa* from China. The mitochondrial genome of E. fossa is 15892 bp in length, exhibiting high AT-rich bias (75.2%). It includes 13 protein-coding genes, 2 ribosomal RNA genes, and 22 transfer RNA genes, consistent with the gene content of other Rhaphidophorinae species.

## ANNOUNCEMENT

The subfamily Rhaphidophorinae (Orthoptera: Rhaphidophoridae) comprises wingless, nocturnal insects distributed from Eurasia to Australasia and notably diverse in China (1). However, taxonomic and genomic studies on this group remain limited, especially for the genus *Eurhaphidophora. Eurhaphidophora fossa* was first reported from Yunnan, China, and is characterized by the trapezoidal process on the male ninth abdominal tergite with a distinct longitudinal furrow on its dorsal surface (2). We sequenced the mitogenome of *E. fossa* to address this gap and resolve its phylogenetic position.

The holotype specimen of *E. fossa* was collected from Bulangshan, Menghai, Yunnan, China (21.6312°N 100.3242°E) in 2019, preserved in absolute ethanol, and stored at −4°C in the Guangxi Normal University (GXNU). Total genomic DNA was extracted from the hind leg muscle tissue using the TIANamp Genomic DNA Kit following the manufacturer’s standard protocol. High-throughput sequencing was performed by Beijing Berry Genomics; a 150-bp paired-end library was built with the MGIEasy Kit, and sequencing was done on an Illumina NovaSeq 6000. Raw sequencing reads were processed using fastp v.0.23.4 (3) by trimming adapter sequences and primer residues and filtering out low-quality reads, those with Phred quality scores <Q5 or containing more than three ambiguous N bases. After filtering, the 42,608,798 high-quality clean reads were retained for assembly. The mitogenome was assembled with NOVOPlasty 4.3.3 (4) using *Rhaphidophora quadrispina* (NC067624) as reference, with parameters set as mito type, 14,000–18,000 bp range, and k-mer 39 (5). This assembly generated a confirmed circular sequence, which was then rotated to start at tRNA-Ile using Geneious Prime 2025.0.2 (6). Annotation was done via the MITOS2 webserver on the Galaxy platform under the invertebrate genetic code and the RefSeq 89 Metazoa standards, with default parameters (7). The initial annotation results were manually checked using MEGA v.11 to verify the start and stop codons of all protein-coding genes and ensure proper gene structure (8). AT/GC skew was calculated using PhyloSuite v1.2.3 (9), and the mitogenome map was generated using Chloroplot 0.2.4 (10).

**Fig 1:**
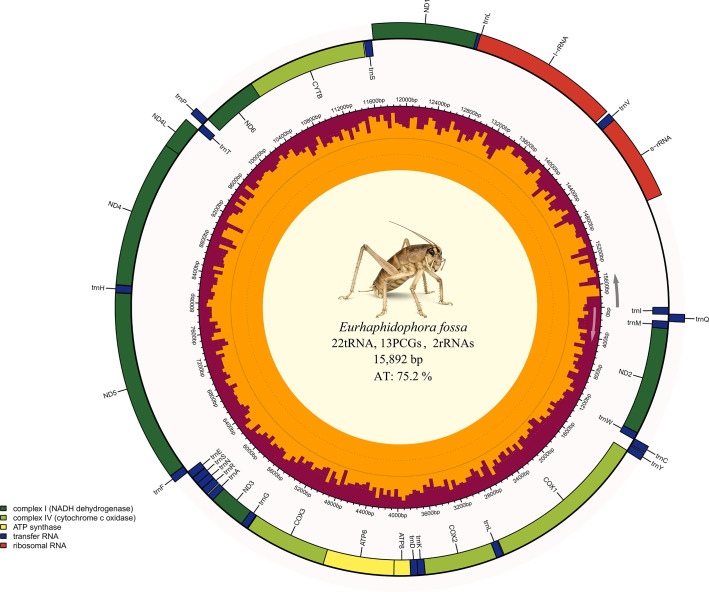
Circular map of the mitochondrial genome of *E. fossa*. The center shows a specimen photo, the inner ring indicates the GC content with arrows marking the transcription direction, and the outer ring shows all annotated genes.

The complete circular mitochondrial genome (GenBank accession number PX412915) of *E. fossa* has a total length of 15,892 bp and an AT content of 75.2% (GC content: 24.8%), which conforms to the conserved feature of high AT bias in insect mitochondrial genomes. It contains 37 genes, including 13 PCGs, 22 tRNA genes, 2 rRNA genes, and 1 non-coding control region (D-loop) (Fig. 1). Table 1 shows that the 13 PCGs predominantly use the typical ATN start codon, with exceptions in COX1 (TAT) and ND1 (TTG). The TTG start codon in ND1 is consistent with that of its closely related species *Rhaphidophora duxiu* (11). Most of the 13 PCGs have the complete TAA stop codon, except for a few with incomplete TA (COX2, ND3, and ND1) or T (ND4L) codons, whose translation may involve polyadenylation to compensate for the defect and ensure termination (12, 13). The lengths of the 22 tRNA genes range from 65 to 72 bp. The rRNA genes include 16S rRNA (1,329 bp) and 12S rRNA (790 bp).

**TABLE 1:** Mitochondrial genome architecture, gene content, and codon usage in *E. fossa*

Gene	Type	Minimum nucleotide position	Maximum nucleotide position	Length	Start codon	Stop codon	Direction
tRNA-Ile	tRNA	1	67	67	–^*a*^	–	Forward
tRNA-Gln	tRNA	64	133	70	–	–	Reverse
tRNA-Met	tRNA	132	201	70	–	–	Forward
ND2	CDS	202	1,230	1,029	ATG	TAA	Forward
tRNA-Trp	tRNA	1,232	1,300	69	–	–	Forward
tRNA-Cys	tRNA	1,292	1,356	65	–	–	Reverse
tRNA-Tyr	tRNA	1,365	1,431	67	–	–	Reverse
COX1	CDS	1,424	2,965	1,542	ATT	TAA	Forward
tRNA-Leu	tRNA	2,974	3,041	68	–	–	Forward
COX2	CDS	3,042	3,734	693	TAT	TAG	Forward
tRNA-Lys	tRNA	3,735	3,805	71	–	–	Forward
tRNA-Asp	tRNA	3,804	3,872	69	–	–	Forward
ATP8	CDS	3,873	4,037	165	ATT	TAA	Forward
ATP6	CDS	4,031	4,708	678	ATG	TAA	Forward
COX3	CDS	4,708	5,496	789	ATG	TAA	Forward
tRNA-Gly	tRNA	5,503	5,570	68	–	–	Forward
ND3	CDS	5,571	5,924	354	ATT	TAG	Forward
tRNA-Arg	tRNA	5,922	5,986	65	–	–	Forward
tRNA-Ala	tRNA	5,999	6,068	70	–	–	Forward
tRNA-Asn	tRNA	6,070	6,136	67	–	–	Forward
tRNA-Ser	tRNA	6,136	6,203	68	–	–	Forward
tRNA-Glu	tRNA	6,207	6,277	71	–	–	Forward
tRNA-Phe	tRNA	6,275	6,342	68	–	–	Reverse
ND5	CDS	6,344	8,074	1731	ATT	TAA	Reverse
tRNA-His	tRNA	8,077	8,144	68	–	–	Reverse
ND4	CDS	8,148	9,483	1,336	ATG	TAA	Reverse
ND4L	CDS	9,477	9,770	294	ATG	T	Reverse
tRNA-Thr	tRNA	9,772	9,837	66	–	–	Forward
tRNA-Pro	tRNA	9,837	9,904	68	–	–	Reverse
ND6	CDS	9,907	10,434	528	ATT	TAA	Forward
CYTB	CDS	10,434	11,570	1,137	ATG	TAA	Forward
tRNA-Ser	tRNA	11,582	11,651	70	–	–	Forward
ND1	CDS	11,668	12,618	951	TTG	TAG	Reverse
tRNA-Leu	tRNA	12,618	12,687	70	–	–	Reverse
16S rRNA	rRNA	12,647	13,975	1,329	–	–	Reverse
tRNA-Val	tRNA	14,002	14,073	72	–	–	Reverse
12S rRNA	rRNA	14,073	14,862	790	–	–	Reverse

^*a*^ –, not applicable.

## Data Availability

The complete mitochondrial genome sequence of *Eurhaphidophora fossa* is available in GenBank under accession number (PX412915). The associated BioProject, SRA, and BioSample numbers are PRJNA1335411, SRR35895472, and SAMN51987872. The mitochondrial genome referenced in the text is *Rhaphidophora quadrispina*. GenBank accession number NC067624.
